# An older diabetes-induced mice model for studying skin wound healing

**DOI:** 10.1371/journal.pone.0281373

**Published:** 2023-02-17

**Authors:** Carlos Poblete Jara, Guilherme Nogueira, Joseane Morari, Thaís Paulino do Prado, Renan de Medeiros Bezerra, Lício A. Velloso, William Velander, Eliana Pereira de Araújo

**Affiliations:** 1 Department of Chemical and Biomolecular Engineering, University of Nebraska-Lincoln, Lincoln, NE, United States of America; 2 Faculty of Medical Sciences, University of Campinas, Campinas, Brazil; 3 Laboratory of Cell Signaling, Obesity and Comorbidities Research Center, University of Campinas, Campinas, Brazil; 4 University of Campinas, Campinas, Brazil; 5 Faculty of Nursing, University of Campinas, Campinas, Brazil; Indiana University Purdue University at Indianapolis, UNITED STATES

## Abstract

Advances in wound treatment depend on the availability of animal models that reflect key aspects of human wound healing physiology. To this date, the accepted mouse models do not reflect defects in the healing process for chronic wounds that are associated with type two diabetic skin ulcers. The long term, systemic physiologic stress that occurs in middle aged or older Type 2 diabetes patients is difficult to simulate in preclinical animal model. We have strived to incorporate the essential elements of this stress in a manageable mouse model: long term metabolic stress from obesity to include the effects of middle age and thereafter onset of diabetes. At six-weeks age, male C57BL/6 mice were separated into groups fed a chow and High-Fat Diet for 0.5, 3, and 6 months. Treatment groups included long term, obesity stressed mice with induction of diabetes by streptozotocin at 5 months, and further physiologic evaluation at 8 months old. We show that this model results in a severe metabolic phenotype with insulin resistance and glucose intolerance associated with obesity and, more importantly, skin changes. The phenotype of this older age mouse model included a transcriptional signature of gene expression in skin that overlapped that observed with elderly patients who develop diabetic foot ulcers. We believe this unique old age phenotype contrasts with current mice models with induced diabetes.

## Introduction

Diabetes mellitus (DM) is a chronic metabolic disease characterized by hyperglycemia that results from deficiencies in insulin secretion and/or action. It is one of the major global health problems affecting over 463 million people worldwide and projecting an increase to 578 million by the end of 2023 [1]. Several genes of the major histocompatibility complex have been described against the insulin pathway in Type 1 diabetes [2]. However, the vast majority of cases are Type 2 diabetic patients as a multifactorial result of insulin resistance and lifestyle imbalance [3]. As a chronic and refractory disease, DM affects every tissue and organ in the body, including the skin. Studies show that up to two-thirds of diabetic patients have skin complications at some point during their lifetime [4]. There are several mechanisms behind DM-associated skin abnormalities, which include, but are not restricted to abnormal regulation of inflammatory products, impaired angiogenesis, and impaired growth factor production [5].

During the course of DM, two features seem central to explain the risk for developing of skin function failures: i, an accelerated skin aging [4, 6] and ii, chronic inflammatory state, which is particularly accentuated in diabetic foot ulcers (DFU) [7]. DFU is the most common complication affecting DM patients; it increases the risk for development of osteomyelitis, which can lead to lower extremity amputations [8, 9]. Treating DFU has proven difficult as it results from a complex pathophysiology [9]. Moreover, due to ethical concerns, trials for new therapeutic interventions in humans are limited, which delays the development of effective strategies. Therefore, testing new approaches to treat DFU must rely, at least during early phases, on the existence of appropriate experimental models.

Over the years, several experimental rodent models have been used in studies aimed at evaluating interventions for treating DFU [10, 11]. These include spontaneous autoimmune DM, genetically induced DM models; diet-induced DM models and pharmacologically induced DM models [12–14]. Regardless of the benefits obtained from each of these models, they all have particular limitations. One important limitation that occurs in virtually all models employed to date relies on the fact that they do not exhibit simultaneously two important features that are common to most patients with DFU; severe metabolic dysregulation and aged skin. Ideally, an experimental model for studying DFU should have close similarities to the clinical and pathological landscape of human DFU. In this study, we describe a model in which DM and aging result in skin alterations that display similarities with those found in humans. Previous reports [15] identified that db/db mice wound model (mutation in the leptin receptor) exhibit severe impairment in wound healing compared to the streptozotocin-induced C57BL/6J mice model. However, it is well described that genetic mutations are not the central cause of the inflammatory syndrome that impairs wound healing observed in type 2 human diabetes. In this way, the clinically relevance of a mutant model is limited for human comparison. Our hypothesis is that long-term exposure to physiologic stress caused by metabolic syndrome it is a central cause of the inflammatory syndrome that impairs wound healing observed in Type 2 human diabetes. The present study will show that long-term physiologic stresses can result in an inflammatory syndrome with impaired wound healing.

## Results

### Diabetes induction

**Fig 1:**
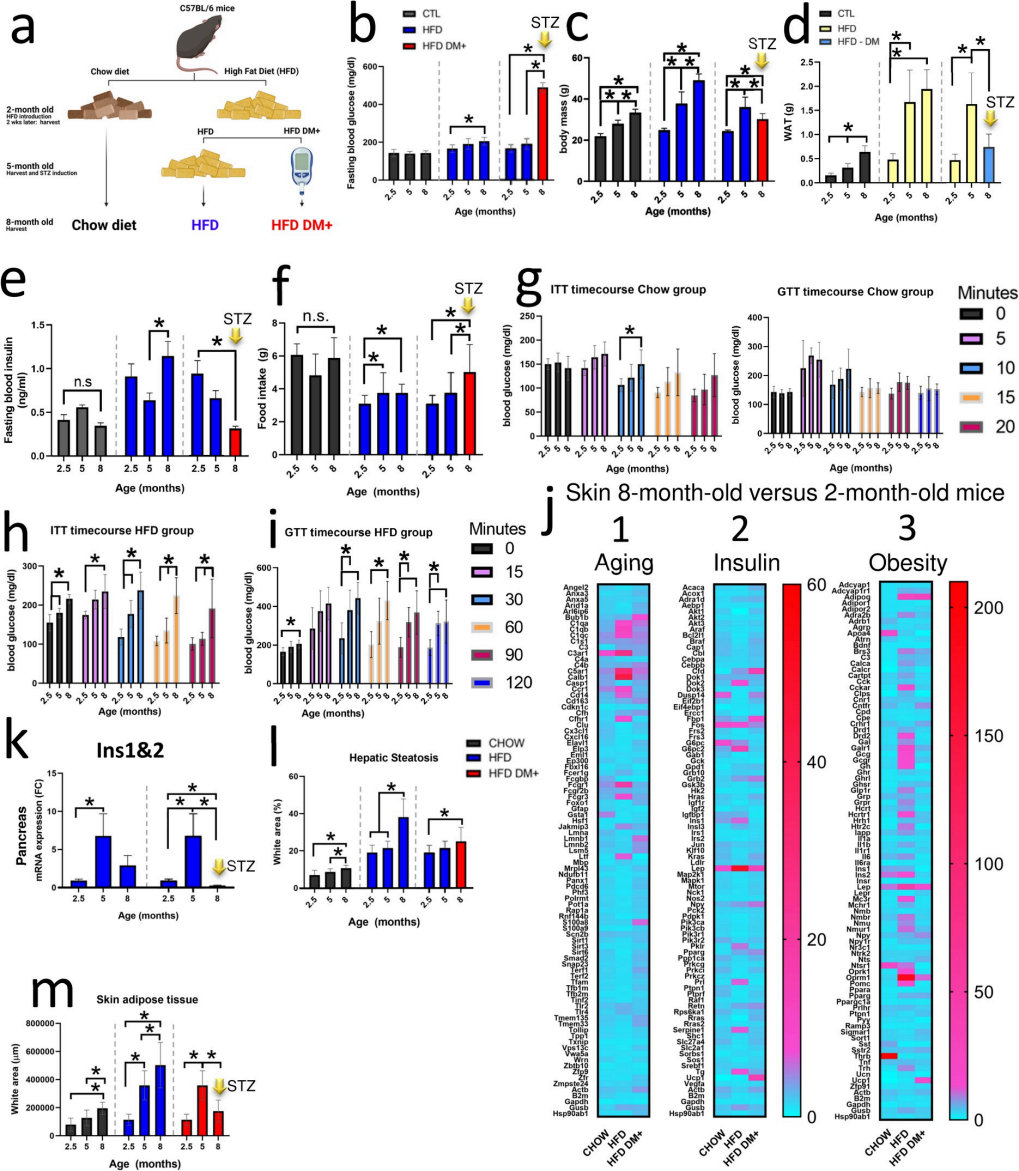
Aging comparison. Schematic representation of experimental design. C57BL/6 mice were fed either with chow Diet or High Fat diet. Some 5-month-old HFD animals were treated with streptozotocin for Diabetes induction (HFD DM+ group) generating two HFD groups: a) HFD DM+ (T2DM-like) and b) HFD non-diabetic **(a)**. Fasting blood glucose in mg/mL **(b)**, body mass in grams **(c)**, White adipose tissue grams **(d)**, Fasting blood insulin in ng/mL **(e)**, food intake in grams, as well as Insulin and Glucose tolerance testing (mg/mL) **(g-i)** at 2.5, 5 and 8-month old of chow, HFD and HFD DM+ animals. Three different mRNA arrays (Aging, Insulin and Obesity) of dorsal skin samples from 2 and 8-month old mice. 2-month old mice were considered as Fold Change references **(j)**. mRNA expression in Fold change of *Ins1* and *Ins2* average genes (*Ins1&Ins2*) of chow, HFD and HFD DM+ animals (2.5-month old animals as reference) **(k)**. Adipose deposition expressed percentage in liver sections **(l)**. Adipose deposition expressed in area (μm) of skin sections (5-μm thickness) **(m)**. *: p value <0.05. Yellow arrow with “STZ”: after 3 months of streptozotocin injections.

5-month old C57BL/6J mice were fed on HFD for 3 months when five low-dose injections of streptozotocin (STZ) (50 mg/kg, i.p.) were performed (Fig 1A). Our results showed that after four weeks from STZ injections, HFD *diabetic* (HFD DM+) mice increased blood glucose levels up to 500mg/dL (489.7 mg/dL HFD DM+ vs 143 mg/dL age-matched non-diabetic mice, *p value* <0.0001). In addition, our result showed chow diet did not affect fasting glycemia during the 8-month of analysis (Fig 1B, black bars). However, the 8-month old mice fed on HFD increased fasting glycemia compared to diet-paired 2.5-month old group (from 166 mg/dL in young to 206 mg/dL in older mice, *p value* 0.0268) (Fig 1B, blue bars). Moreover, the 8-month old HFD DM+ group increased even more fasting glycemia (3 fold more after 3 months streptozotocin injections) compared to diet-paired 2.5-month old counterparts (from 166 mg/dL younger to 490 mg/dL in older mice, *p value* <0.0001) (Fig 1B, red bars).

#### Aging increases body mass and white adipose tissue deposition in mice

Aging affects body mass (Fig 1C). Independently of food characteristics, mice fed on chow or HFD increased body mass during the 8 months of analysis (Fig 1C and Table 1, *p value* <0.0001). 8-month old mice fed on chow diet increased body mass from 22g (2.5-month old) to 33g at rate 60-80mg/day (S1B Fig). 8-month old mice fed on HFD increased body mass from 25g (2.5-month old) to 49g at rate 130-170mg/day (S1B Fig). However, after streptozotocin injections, HFD DM+ mice decreased body mass from 36g (5-month old) to 30g at a rate of -65 mg/day (S1B Fig).

**Table 1:** Body mass from chow, HFD and HFD DM+ mice during 8 month of analysis.

		Age (months)			
**Body mass**		2.5	5	8	*p value*
**Chow**	Mean (g)	21.9	27.9	33.4	<0.0001
	SD	1.3	1.8	1.7	
**HFD**	Mean (g)	24.8	37.8	49.1	<0.0001
	SD	0.9	5.7	3.1	
**HFD DM+**	Mean (g)	24.4	36.0	30.1	<0.0001
	SD	0.5	4.9	2.7	
	*p value*	<0.0001	0.0024	<0.0001	

ANOVA *p value*.

In addition, we found increased Epididymal White Adipose Tissue (WAT) mass in older 5- and 8-month old mice fed with chow diet compared to younger 2.5-month old mice fed on chow (from 0.16g to 0.64g, *p value* <0.0001). This increase represents a 4-fold WAT mass in the 8-month old group fed with chow diet compared with their younger counterpart (Fig 1D, black bars). We found this WAT mass increased following the pattern of the body mass (S1A Fig). Additionally, mice fed on HFD increased even more WAT mass (Fig 1D, yellow bars). We found increased WAT in older 8-month old animals fed with HFD compared with younger HFD 2.5-month and 5-month old groups (from 0.48g to 1.94g, *p value* 0.0001). The increased WAT mass represents 4-fold the WAT mass of younger mice fed on HFD (Fig 1D). As well as the chow group, we found this increase in WAT mass also follows the body mass pattern (S1A Fig).

However, the biggest differences were found in older 8-month-old HFD DM+. HFD DM+ mice decreased WAT mass compared to younger diet-paired 5-month-old mice (from 1.7g to 0.7g, *p value* 0.0005) representing 2.3-fold less WAT mass (Fig 1D, blue bar). Interestingly, over the course of 3 months with induced diabetes beginning at 5 months’ age, this decrease in WAT mass also follows the body mass decline pattern of the HFD DM+ animals (S1A Fig).

#### The hyperglycemic phenotype includes both insulin deficiency and polyphagia in HFD DM+

We explored how aging affects insulin levels and polyphagia (Fig 1E–1F). In 2.5-month-old mice fed on chow diet showed 0.4 ng/mL insulin levels while 2.5-month-old HFD mice showed 0.9 ng/mL insulin levels with no polyphagia. In 8-month-old mice fed on chow diet showed 0.35 ng/mL insulin levels while 8-month-old mice fed on HFD presented as 1.1 ng/mL insulin levels with no polyphagia. However, 8-month HFD DM+ presented 0.3ng/mL insulin levels with polyphagia.

8-month-old HFD mice increased food intake compared to 2.5-month-old HFD mice (from 3g/day to 4g/day, *p value* 0.0232, Fig 1F). 8-month-old HFD DM+ mice further increased food intake to 5g/day (*p value* <0.0001, Fig 1F). In contrast, 8-month-old Chow diet mice showed no differences in food intake (Fig 1F).

#### Aging impacts Insulin and Glucose tolerance in mice fed on HFD diet

We investigated the contribution of aging to insulin sensitivity by a) intraperitoneal injection of insulin (ITT) and b) glucose metabolism by intraperitoneal injection of glucose (GTT) in chow, and HFD mice. Our results showed aging has a progressive and detrimental effect on insulin sensitivity and glucose metabolism in 8-month-old HFD mice (Fig 1H and 1I). The increased glucose levels occurred at 15, 30, 60, 90 and 120 min post insulin injection compared to 2.5-month old HFD mice (*p value* <0.05, Fig 1H). GTT results in 8-month HFD mice also showed increased glucose levels 30 min after glucose challenge compared to 2.5-month HFD mice (*p value* <0.05, Fig 1I). Our results in 2.5, 5 and 8-month old chow fed mice showed no differences in the clearance pattern of plasma glucose except after 10 min post insulin injection (from 106 mg/dL to 150 mg/dL, *p value* 0.0260, Fig 1G left).

#### Aging impacts gene expression of insulin pathway, obesity and aging associated genes

To better understand the effect of aging in the skin of chow, HFD and HFD DM+ mice, we screened three different gene arrays: Aging, Insulin Pathway and Obesity.

*Column 1*. Fig 1J shows the expression of age-associated genes in the skin of 8-month-old animals relative to 2-month-old counterparts. 8-month-old chow animals overexpressed *C3ar1* (8-fold), *Gsta1* (5-fold), and *Ccr1* (4-fold). In addition, 8-month old HFD animals overexpressed *Calb1* (42-fold), *C5ar1* (30-fold), and *C3ar1* (26-fold). Skin from 8-month-old HFD DM+ mice overexpressed *Bub1b* (7-fold), *S100a8* (6-fold), and *Lmnb1* (5-fold).

*Column 2*. Fig 1J shows the expression of Insulin-associated genes in the skin of 8-month-old animals relative to 2-month-old counterparts. 8-month-old chow animals overexpressed *Lep* (9-fold), *Fos* (9-fold), and *G6pc* (8-fold), the HFD animals overexpressed *Lep* (50-fold), *G6pc2* (15-fold), and *Fos* (7-fold), and the HFD DM+ overexpressed *Bub1b* (7-fold), *S100a8* (6-fold), and *Lmnb1* (5-fold).

*Column 3*. Fig 1J shows the expression of Obesity-associated genes in the skin of 8-month-old animals relative to 2-month-old counterparts. 8-month-old chow animals overexpressed *Thrb* (207-fold), *Ntsr1* (20-fold), and *Apoa4* (16-fold), the HFD animals overexpressed *Oprm1* (182-fold), *Lep* (48-fold), and *Adipoq* (25-fold), and the HFD DM+ overexpressed *Adipoq* (28-fold), *Ucp1* (15-fold), and *Lep* (13-fold).

#### Aging affects pancreatic Insulin 1 and 2 expressions in mice fed on HFD

*Ins1* and *Ins2* genes encode for insulin 1 and 2, peptides that are vital in the regulation of carbohydrate and lipid metabolism. Our result showed that total *Ins1&2* gene expression increased in **pancreatic** tissue in 5-month-old mice fed on HFD (Fig 1K, left). After streptozotocin injections, the HFD DM+ mice decreased pancreatic total *Ins1&2* gene expression (Fig 1K, right).

#### Aging affects the accumulation of intrahepatic and dermal fat in HFD and chow fed mice

Both, the accumulation of intrahepatic fat (hepatic steatosis) and insulin resistance are associated with liver metabolic dysfunction [16, 17]. For this reason, we explored the intrahepatic fat percentage in mice fed on chow, HFD and HFD DM+. Fig 1L shows the histological percentage of intrahepatic fat. After 8 months on chow diet, intrahepatic fat increased from 7% to 11% when comparing to 2-month old mice (*p value* <0.0001). 8-month old HFD mice had increased intrahepatic fat percentage from 19% to 38% (*p value* <0.0001). HFD DM+ animals increased from 19% to 25% (*p value* 0.0148).

Dermal white adipose tissue (dWAT), occurs in the dermis underlying the reticular dermis [18], and participates in thermogenesis, wound healing and immune defense against infection [19]. We investigated dermal fat deposition in mice fed on chow, HFD and HFD DM+. Fig 1M shows the histological presence of dWAT. 8-month-old chow and HFD animals increased dWAT relative to both 2- and 5-month old mice (*p value* <0.0001). In contrast, HFD DM+ mice decreased dWAT relative to 5-month-old HFD mice (*p value* <0.0001).

#### HFD and hyperglycemia affects body mass in HFD DM+ mice

**Fig 2:**
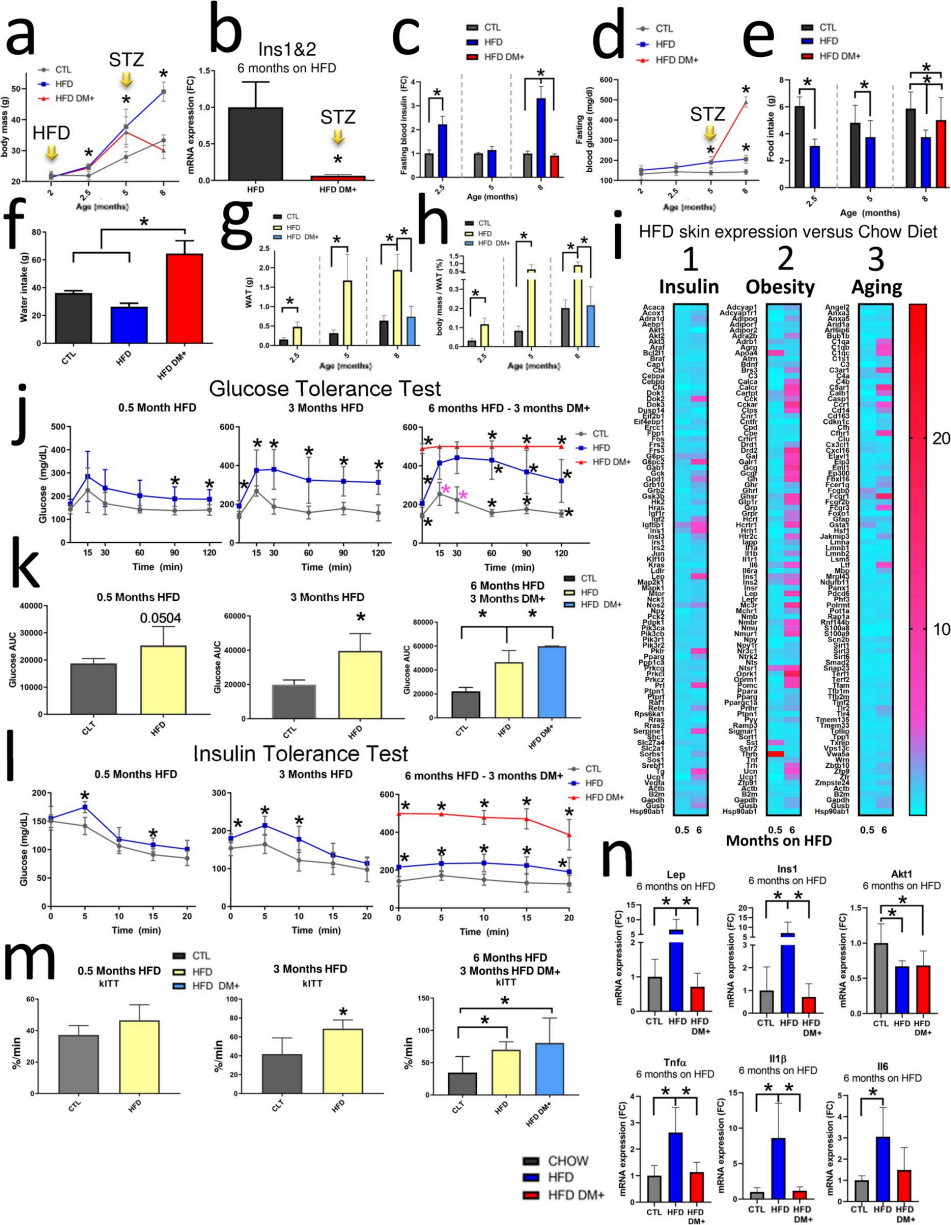
Diet comparison. body mass in grams of chow (Control), HFD, and HFD DM+ until 8 months age **(a)**. Pancreatic *Ins1* and *Ins2* average expression (*Ins1&2*), HFD mice as Fold change reference **(b)**. Fasting insulin protein levels expressed from ELISA experiments. chow animals (Control) were considered the Fold Change (FC) reference **(c)**. Blood glucose expressed in mg/dL in fasting mice after months on chow (Control) and HFD **(d)**. Diet effect in mice Food intake after months on chow (Control) and HFD. Values are daily food intake measurement during one week of follow-up (**e)**. Diet effect in mice Water intake after months on Chow (Control) and HFD. Values are daily water intake values during one week of follow-up (**f)**. Diet effect in Epididymal White Adipose Tissue (WAT) deposition in grams **(g)** and body mass normalized **(h)**. Different mRNA arrays (Aging, Obesity and Insulin) of dorsal skin samples from mice fed on HFD for 0.5 and 6 months. chow animals were considered Fold Change references **(i)**. Glucose and Insulin tolerance test performed in fasting mice after 0.5, 3 and 6 months on HFD. HFD DM+ (red line) spent 3 months hyperglycemic (**j, l**). Area Under the curve (AUC) of 0.5, 3 and 6 months on HFD compared to age-matched controls. HFD DM+ (light blue bar) spent 3 months hyperglycemic **(k)**. The rate constant for glucose disappearance during insulin tolerance test (kITT) of 0.5, 3 and 6 months on HFD **(m)**. mRNA expression in Fold change of *Lep*, *Ins1*, Akt1, Tnfα, Il1β and Il6 of chow, HFD and HFD DM+ animals (chow diet animals as reference) **(n)**. Yellow arrow with “STZ”: day of streptozotocin injections. *: p value <0.05.

In our previous studies of mice with less than 5-month age, we have shown that HFD affects body mass [20–23]. Fig 2A shows that after only 2 weeks on HFD, mice had 13% increased body mass as compared to the chow diet group (24.6g HFD vs 21.9g chow, *p value* <0.0001). 3 months additional feeding on HFD resulted an increase of 35% of body mass compared to chow diet (37.8g HFD vs 27.9g chow, *p value* 0.0024). A total of 6 months on HFD increased body mass by 47% compared to chow diet (49.1g vs 33.5g, *p value* <0.0001). In contrast, HFD DM+ mice after a total of 6 months on HFD had decreased 39% body mass compared with their non-diabetic HFD counterparts (30.1g HFD DM+ vs 49.1g HFD, *p value* <0.0001).

#### HFD DM+ have both decreased insulin gene expression and circulating insulin levels

Our result showed that after streptozotocin treatment (STZ), HFD DM+ mice decreased Pancreatic *Ins1* and *Ins2* expression (0.05-fold change, *p value* 0.0335) at 8 months of age (Fig 2B).

Mice fed for only 0.5 months on HFD beginning at 2 months age had 2.2-fold increased fasting blood insulin levels compared to chow animals (1.3ng/mL vs 0.41ng/mL, *p value* 0.0374, Fig 2C). Mice after a total of 6 months on HFD increased 3.3-fold insulin levels to 1.1ng/mL while chow animals had 0.3 ng/mL (*p value* 0.0017). 8-month HFD DM+ (6 months on HFD) showed 0.3ng/mL (Fig 2C).

We found mice fed on HFD for 3 months increased 37% Fasting blood glucose compared to chow diet mice (190mg/dL vs 139mg/dL, *p value* 0.0021, Fig 2D). After 6 months on HFD, mice had a 41% increased glucose levels compared to chow mice (201mg/dL vs 142mg/dL, *p value* <0.0001). In contrast, HFD DM+ mice had 144% and 243% increased Fasting blood glucose when compared with HFD- and chow, respectively (490mg/dL vs 201mg/dL vs 142mg/dL, *p value* <0.0001).

#### Diabetic mice showed polyphagic food intake and polydipsic water intake

Our results showed HFD DM+ mice increased daily Food intake (Fig 2E). HFD DM+ mice displayed a 25% increased daily Food intake compared with their same-age non-diabetic HFD counterparts (5g vs 4g, *p value* 0.0021). HFD DM+ showed *polydipsic* water intake compared either to chow diet or their non-diabetic HFD counterparts (16g vs 9g, *p value* <0.0001) (Fig 2F). This difference in diabetic mice represents a 44% more daily water intake compared to the HFD mice and 63% more water intake compared to mice fed on chow diet.

#### HFD DM+ have decreased epididymal white adipose

HFD mice increased WAT mass compared to chow diet animals (480mg vs 156mg, *p value* 0.0002) (Fig 2G). This difference represents a 207% more WAT mass in HFD animals compared to the chow group. An additional 3 months on HFD resulted a further 431% increase of WAT (1674mg vs 315mg, *p value* 0.0007) compared to age-paired chow diet animals (Fig 2G). After 6 months on HFD, mice further increased WAT mass by 205% (1942mg vs 638mg, *p value* <0.0001) compared to chow diet (Fig 2G). In contrast, 8-month HFD DM+ mice decreased WAT mass by 161% compared to their non-diabetic same-age HFD counterparts (743mg vs 1942mg, *p value* <0.0001) (Fig 2G). We identified similar results of White adipose tissue mass after body mass correction (Fig 2H).

#### HFD and HFD DM+ mice present chronic glucose intolerance

GTT results showed increased plasma glucose levels at 90 min (138mg/dL vs 189mg/dL) and 120 minutes (140mg/d vs 187mg/dL) after 0.5 months of HFD compared with chow diet mice (2.5-month-old, *p value* <0.05) (Fig 2J). After 3 months of HFD feeding, mice increased plasma glucose levels at each time point of GTT sampling (0, 15, 30, 60, 90 and 120 min) (5-month-old, *p value* <0.05). 8-month-old HFD mice (HFD for 6 months) also had increased plasma glucose levels at all times of GTT sampling (*p value* <0.0001). HFD DM+ mice presented higher plasma glucose levels (498mg/dL vs 364mg/dL average) at all GTT time points compared to their age-matched HFD group (*p value* <0.0001, Fig 2J). There were no differences in the AUC (from plasma glucose levels) of animals fed on HFD for 0.5 months (Fig 2K). After 3 months on HFD, the AUC increased 99% compared to chow animals (*p value* 0.0010) (Fig 2K). HFD DM+ mice also showed a 170% AUC increase relative to the chow diet group (*p value* <0.0001) (Fig 2K).

#### HFD and HFD DM+ mice present chronic insulin resistance

To determine the whole-body sensitivity to insulin, we measured blood glucose levels after intraperitoneal insulin administration (Fig 2L and 2M). ITT results showed that after 0.5 months on HFD, plasma glucose levels increased at 5 min (175mg/dL vs 142mg/dL) and 15 min (108mg/dL vs 91mg/dL) compared to mice fed on chow diet (*p value* <0.05) (Fig 2L). After 3 months on HFD the plasma glucose levels increased at 0 min (180mg/dL vs 153mg/dL), 5 min (214mg/dL vs 164mg/dL), and 10 min (177mg/dL vs 122mg/dL) (*p value* <0.05). After 6 months on HFD, the plasma glucose levels were increased at all-time points (*p value* 0.0001). 8-month old HFD DM+ mice (6 months on HFD) also presented higher plasma glucose levels at all-time points (*p value* <0.0001). A constant rate of glucose disappearance was observed (Fig 2M) where higher values indicate greater tissue insulin resistance [24, 25]. As results, no kITT differences were observed after 0.5 months of HFD feeding (*p value* 0.0783) (Fig 2M) but kITT increased after 3 months on HFD (*p value* 0.0071) (Fig 2M). This kITT difference represents a 41% more in insulin resistance in HFD animals compared to the chow group. HFD DM+ showed increased kITT values compared to age-matched Chow diet groups (*p value* 0.0274), but not when compared to the HFD group (Fig 2M). This kITT difference in HFD DM+ mice represents a 133% increase in insulin resistance compared to chow diet mice but no difference with the HFD group (p valor 0.7867) (Fig 2M).

#### Diet and glycemia impact on gene expression of insulin pathway, obesity and aging associated genes

*Column 1*. Fig 2I shows the expression of insulin-associated genes in the skin of 8-month old HFD animals relative to 8-month old chow counterparts. After 2 weeks on HFD, skin tissue increased *Igfbp1* (3.2-fold), *G6pc* (2.5-fold), and *Ins1* (2-fold) more transcripts than their age-matched chow diet counterparts. After 6 months on HFD, skin tissue overexpressed *G6pc2* (8.7-fold), *Lep* (8.5-fold), and *Ins1* (6 fold) more transcripts as well as downregulation of Akt1 (0.01 fold) when compared to their age-matched chow diet counterparts (Fig 2I).

*Column 2*. Fig 2I shows the expression of obesity-associated genes in the skin of 8-month old HFD animals relative to 8-month old chow counterparts. After 2 weeks on HFD, skin tissue increased *Thrb* (26-fold), Apoa4 (8.5-fold), and *Sst* (4-fold). After 6 months on HFD, skin tissue overexpressed *Oprk1* (17-fold), Drd2 (11-fold), and *Mc3r* (11-fold) (Fig 2I).

*Column 3*. Fig 2I shows the expression of aging-associated genes in the skin of 8-month old HFD animals relative to 8-month old chow counterparts. After 2 weeks on HFD, only 3 of 89 genes were >2-fold expressed: *Fcgbp* (3-fold), *Gsta1* (2.8-fold), and *Fcgr1* (2.3-fold) had increased expression. After 6 months on HFD, skin tissue from HFD mice increased expression of *Fcgr1* (16-fold), *C5ar1* (11-fold), and *C3ar1* (9-fold) (Fig 2I).

To confirm the above array results from skin samples, we assessed individual gene expression by PCR. Our PCR results showed that 8-month-old mice (6 months on HFD) increased expression of *Lep* (5.6-fold) as compared to chow diet mice (*p value* 0.0011) and also 5.9 fold more *Lep* expression compared to HFD DM+ (*p value* 0.0011). After 6 months on HFD, these mice increased 11.2-fold of *Ins1* as compared to chow diet mice (*p value* 0.0003), and 10-fold as compared to HFD DM+ (*p value* 0.0001). *Akt1* was downregulated in both HFD and HFD DM+ mice (*p value* 0.0291). The inflammatory gene markers after, mice increased *Tnfɑ* (*p value* 0.0008), *Il1β* (*p value* 0.0004) and *Il6* (*p value* 0.0156) after 6 months on HFD (Fig 2N).

#### HFD and hyperglycemia affect intrahepatic fat deposition in mice

**Fig 3:**
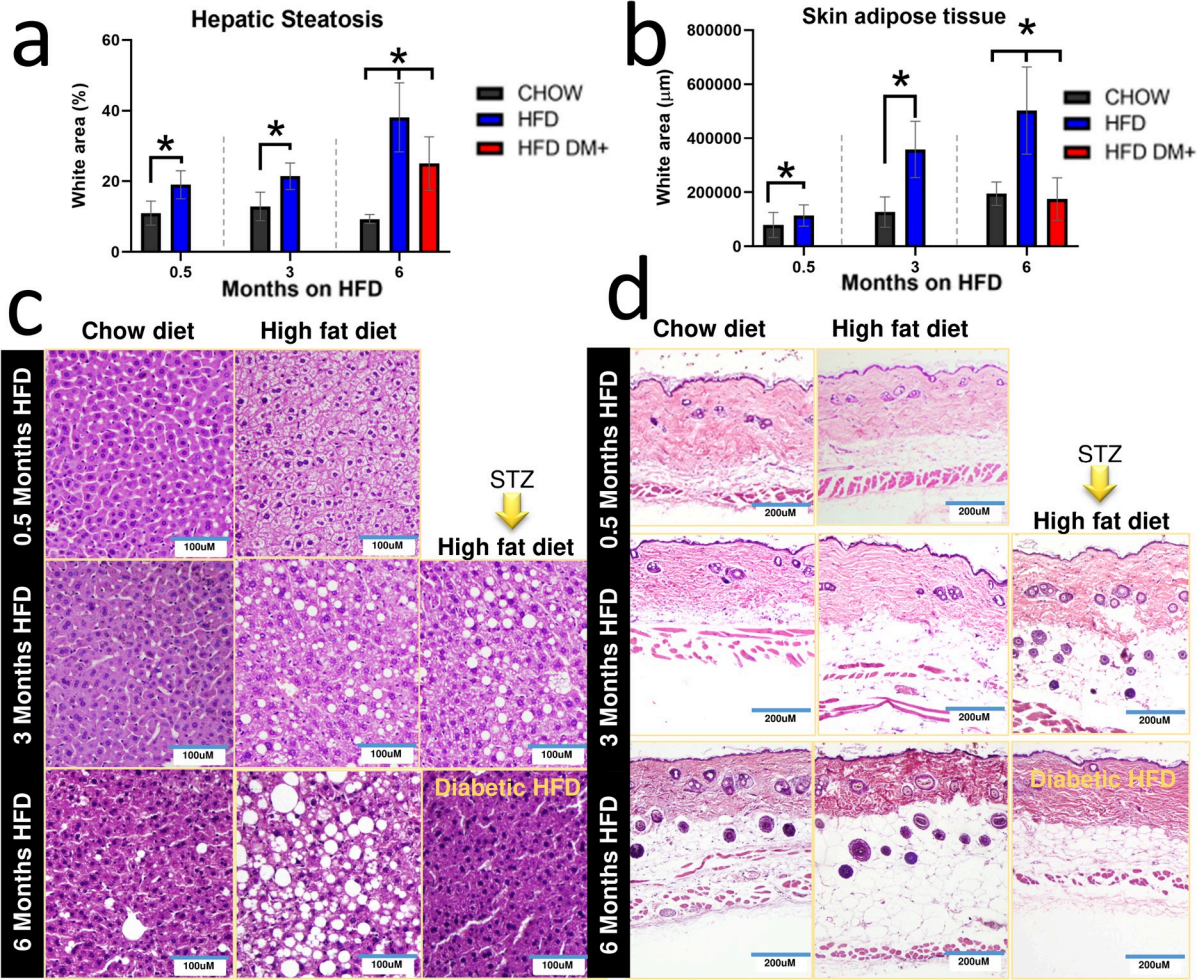
White adipose tissue. Indirect hepatic steatosis was determined by white area quantification (percentage of total section) in 5μm liver sections (triplicate in five different animals). Hepatic steatosis of mice after months on chow (Control) and HFD (**a, c**). Indirect dermal adipose deposition was determined by white area quantification (micrometers of adipose layer under dermis and above muscle layers) in 5μm skin sections (triplicate in five different animals). Skin adipose tissue of mice after months on chow (Control) and HFD (**b, d**).

Our results showed that HFD and hyperglycemia both are associated with accumulation of intrahepatic fat percentage in mice (Fig 3A and 3C). Mice fed for 0.5 months on HFD had increased intrahepatic fat compared to age-matched chow fed mice (19% vs 11%, *p value* <0.0001). Mice fed for 3 months on HFD also had increased intrahepatic fat compared to age-matched chow fed mice (21% vs 13%, *p value* <0.0001). After 6 months on HFD, mice further increased intrahepatic fat compared to age-matched chow fed mice (38% vs 9.3%, *p value* <0.0001). In contrast, HFD DM+ had decreased intrahepatic fat percentage as compared to age-matched HFD fed mice (38% vs 25%, <0.0001) (Fig 3A and 3C).

#### HFD and hyperglycemia affect dermal fat deposition in mice

Our histological analysis showed that HFD and HFD DM+ had different levels of accumulated dWAT (Fig 3B and 3D). Both mice with 0.5 months and 3 months on HFD had increased dWAT as compared to the chow fed mice (*p value* <0.05). In contrast, HFD DM+ mice had decreased dWAT as compared to HFD group (*p value* <0.0001, Fig 3B and 3D).

#### A comparison of gene expression of diabetic human and HFD DM+ mice in skin

**Fig 4:**
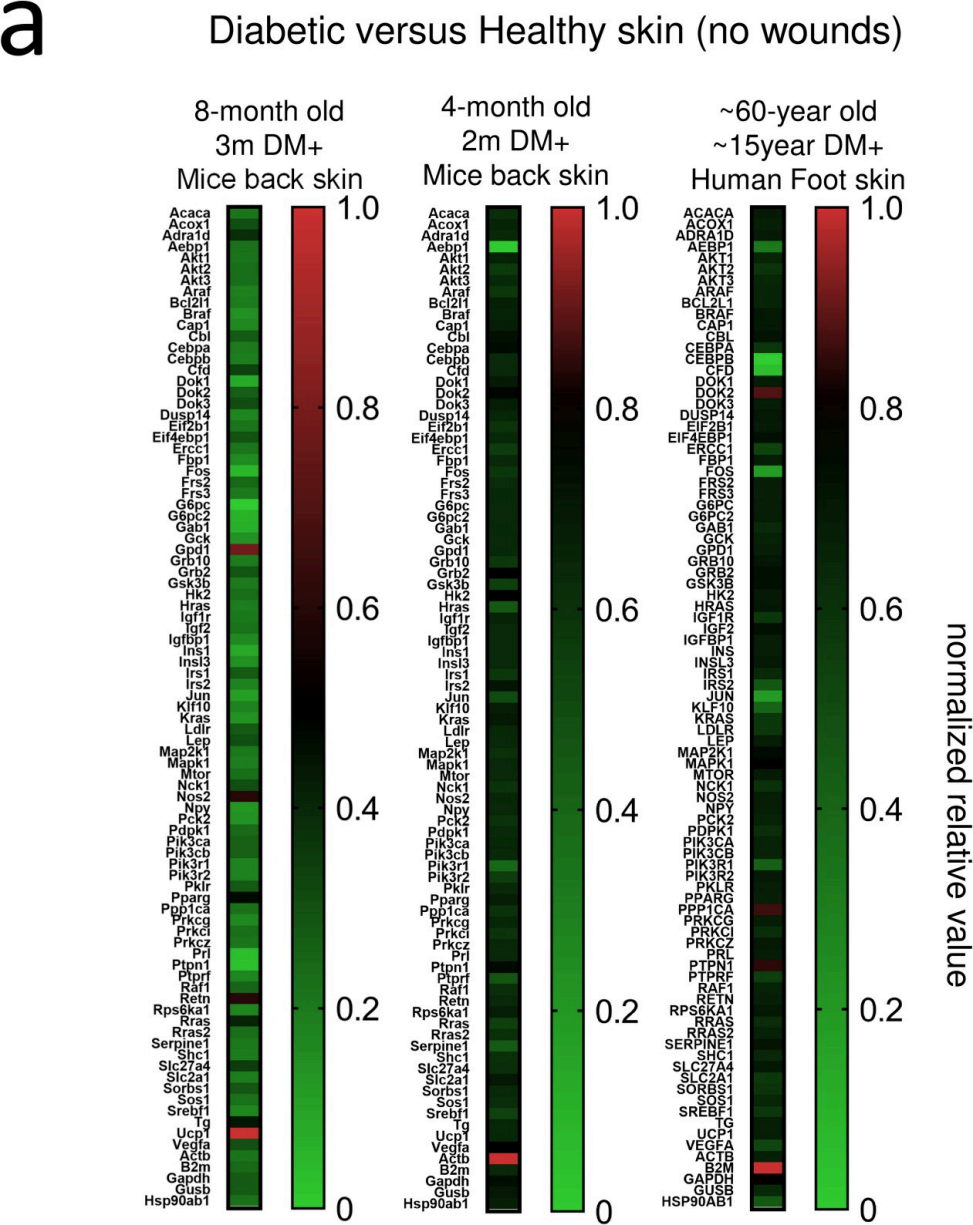
Human and mice skin transcriptomic. Single-cell RNA-seq Pseudobulk analysis from mice and human using as gene list the Mouse Insulin Array. Healthy (non-diabetic, unwounded) was used as reference. Diabetic 4 and 8-month old mice as well as 60 years old human “bulk” normalized gene expression **(a)**.

A Mouse Insulin Pathway array (Qiagen, PAMM-030Z) was used to compare 20-gene expression of skin samples with diabetic human and HFD DM+. Relative to non-diabetic skin samples, our analysis identified 10-common genes: *RRAS*, *SLC27A4*, *CFD*, *EIF4EBP1*, *GRB2*, *SORBS1*, *PTPN1*, *GSK3B*, *HRAS* and *BRAF* (Fig 4A).

We compared this 8-month old HFD DM+ model data to previously published data of 4-month old STZ-induced diabetic non-HFD mice. We identified four common genes increased between older and younger STZ-induced diabetic mice: *Pparg*, *Dok3*, *Vega* and *Grb2* (Fig 4A).

### HFD DM+ showed delayed wound healing rate

**Fig 5:**
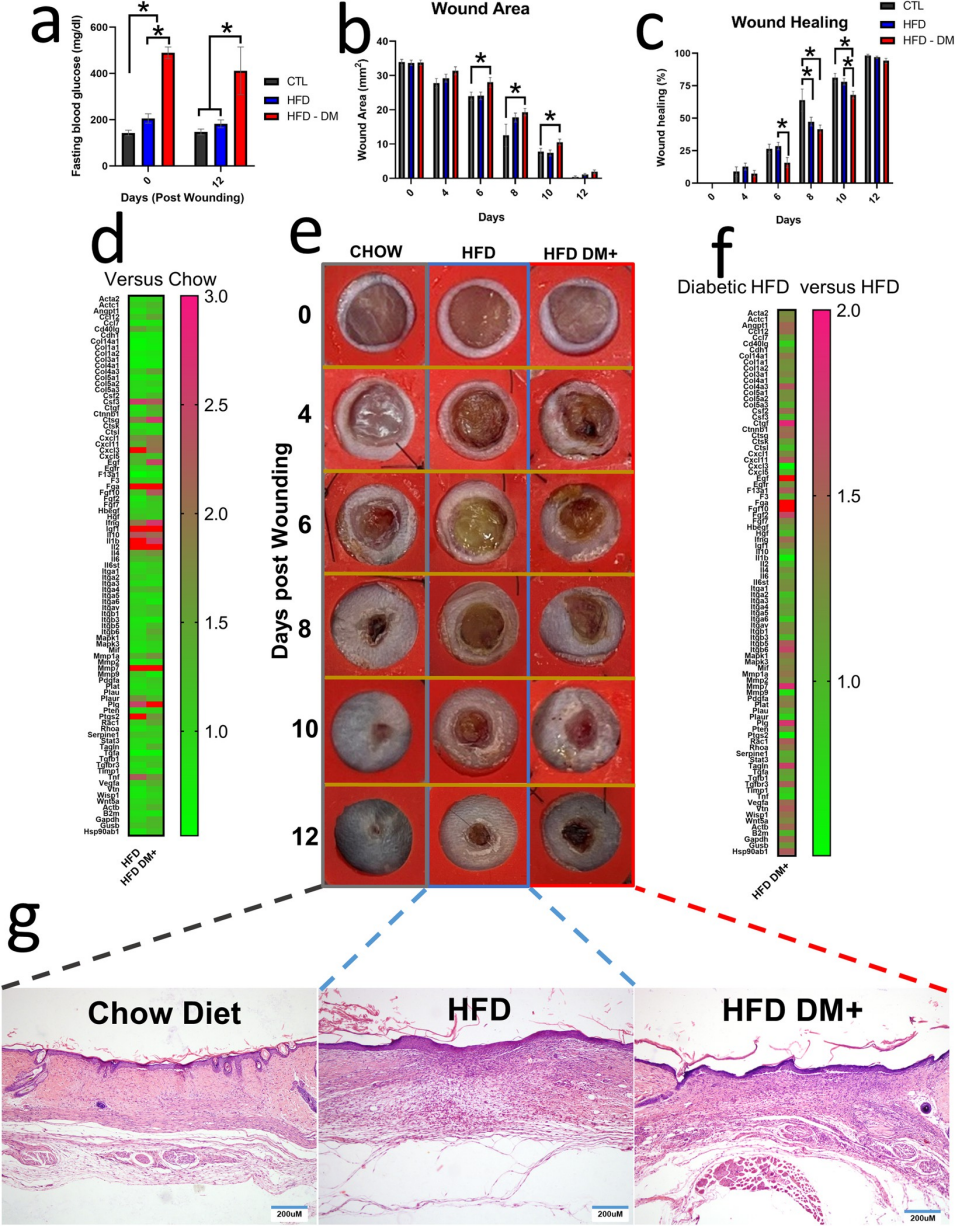
Wound healing process in HFD and HFD DM+ mice. Blood glucose expressed in mg/dL from fasting mice after 0 and 12 days post wounding (a). Wound area expressed in mm2 during 0 to 12 days post wounding (b). Wound healing rate expressed in percentage during 0 to 12 days post wounding (c). mRNA Wound healing array of dorsal wounded skin samples from HFD and HFD DM+ mice. Chow animals were considered Fold Change references (d). Macro documentation of wound healing process **(e)**. mRNA Wound healing array of dorsal wounded skin samples from HFD DM+ mice. HFD animals were used as Fold Change references **(f)**. Micro documentation of wound healing process using skin sections (5 μm) in triplicate from five different mice **(g)**.

We confirmed hyperglycemic status before performing the wound experiment in 8-month old mice. HFD DM+ group presented higher fasting blood glucose compared to chow or HFD mice either the same day of wounding or 12 days post wounding (p value <0.05, Fig 5A).

HFD DM+ mice showed increased wound area compared to chow diet at 6 days (28mm^2^ vs 24mm^2^), 8 days (19mm^2^ vs 13mm^2^), and 10 days (11mm^2^ vs 8mm^2^) post-wounding (p value <0.05, Fig 5B, 5E and 5G). These differences in HFD DM+ mice represent respectively more wound area compared to chow animals and suggested a slower healing process in the HFD DM+ group. Additionally, qualitative analysis suggested HFD and diabetic HFD increased cellularity in 12-day wounded skin tissue (Fig 5G). Microscopically, 8-month-old chow, HFD and HFD DM+ mice showed complete re-epithelialization at 12 days post wounding (Fig 5G).

#### Diet and glycemia affect gene expression of wound healing pathway

Fig 5D shows the expression of wound healing-associated genes in 12 days post wounding skin tissue of 8-month old HFD and HFD DM+ animals relative to 8-month old chow counterparts. After 6 months on HFD, mice increased *Il1β*, *Ptgs2*, *Il10*, *Tnfα*, *Mmp1*, and *Cxcl1* as well as decreased *Tgfa* and *Col14a1* as compared to age-matched chow diet counterparts (p value <0.05). HFD DM+ skin increased *Egf*, *Fgf10*, *Mmp1a*, *Ccl12* as well as downregulation of *Col1a1* when compared to age-matched chow diet counterparts (p value <0.05). Additionally, we explored HFD DM+ gene expression compared to HFD group as baseline. As a result, 12 days post wounding skin tissue increased *Fgf10*, *Egf*, *Ctgf*, *Itgb6*, and *Fgf2*.

**Fig 6:**
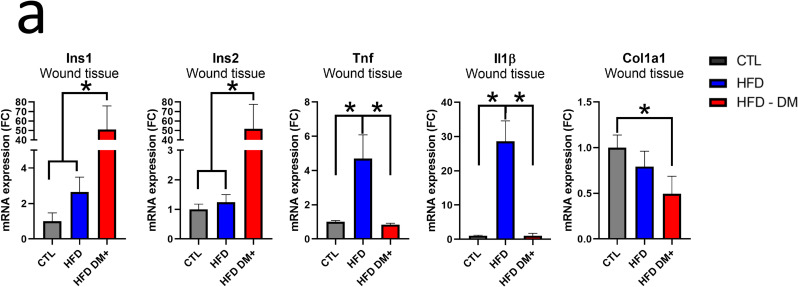
mRNA expression. mRNA expression of *Ins1*, *Ins2*, *Tnfα*, *Il1β* and *Col1a1* from chow, HFD and HFD DM+ animals (chow diet animals as reference) **(a)**.

To confirm the above array results from 12 days post wounded skin samples, we assessed individual gene expression by PCR. Fig 6A showed HFD DM+ increased *Ins1* and *Ins2* as well as decreased *Col1a1* gene expression (p value <0.05). HFD mice increased *Tnfα* and *Il1β* gene expression compared either to chow or HFD DM+ groups (*p value* <0.05).

## Discussion

Here, we described the metabolic phenotype of an experimental model that can be used to study skin wound healing in aging and DM. We showed that an experimental model that associates diet-induced obesity, aging and pharmacological induction of diabetes mellitus results in a severe metabolic phenotype with insulin resistance, reduced b-cell insulin expression and glucose intolerance and this is associated with changes in the skin that match the skin of aging humans with DM.

It is well described that genetic mutations are not the central cause of the inflammatory syndrome that impairs wound healing observed in type 2 human diabetes. Rather it is the long-term exposure to systemic, physiologic stress caused by metabolic syndrome and thereafter the additional long-term exposure to the diabetic condition. The use of genetic mutations in mice in combination with chemical induction of diabetes can cause physiologic stress in several mouse mutation models. It has been suggested that these mouse models likely do not incorporate a sufficiently long exposure to diabetes to emulate the stress seen in the human condition. The novelty of our model differs from these in two important ways: the long-term exposure to metabolic syndrome from a high fat diet and thereafter a long-term exposure to chemically induced diabetes. Our results show that long-term physiologic stresses can result in an inflammatory syndrome with impaired wound healing without genetic mutation. Thus, long term physiologic stress is likely one of the chief risk factors to tissue inflammation and impaired wound healing observed in type 2 diabetes in humans.

Previous studies [15] showed db/db mice wound model exhibit most severe impairment in wound healing compared to the STZ-induced C57BL/6J mice model. However, a recent meta-analysis of 77 studies comparing eight different models of diabetes (678 non-diabetic and 720 diabetic mice) suggested multiple-dose drug-induced diabetes model had the most severe wound healing impairment even when compared to mutant mouse models. Additionally, the meta-analysis showed that longer diabetes period prior to wounding is correlated with greater wound healing impairment [26]. Beside monogenic db/db and Akita (*Ins2* mutation) mice models, new polygenic strain of Type 2 diabetes (NONcNZO10) mutant mice have been described as a new wound healing platform. The NONcNZO10 mutant strain was found to have significant wound healing impairments as well [27]. However, the clinically relevance of a mutant model is limited for human comparison.

DM is a chronic metabolic disease that is associated with accelerated aging resulting in the damage of several tissues and organs in the body [28–30]. The skin is an important target of the metabolic, vascular, immunological, and neural abnormalities that result from a poorly controlled DM. The processes for the development of these problems are not yet entirely elucidated [6, 31]. Diminished lamellar body synthesis, lipid production of epidermal cells, and decreased hydration have also been correlated with persistent hyperglycemia, take the lead to the deterioration of the cutaneous barrier function, and turning the skin prone to dryness and infections [32, 33]. Furthermore, increased oxidative stress and low-grade inflammation are associated with the development of skin lesions from diabetes [34]. Thus, in our animal model, we observed changes in the transcripts of important aging intermediaries and insulin action that could result in alterations in the skin.

Pro-inflammatory cytokines are crucial in regulating the inflammatory process during cutaneous wound healing, that can prolong the inflammatory phase and inhibit healing progression [9, 35, 36]. Furthermore, our results showed higher levels of these cytokines suggesting a prolonged inflammatory phase and delaying the wound healing progression [9, 35, 36]. Similar to a previous studies [7, 37], we observed changes in pro-inflammatory cytokines that are crucial in regulating the inflammatory process during cutaneous wound healing. Specifically, we showed increased *Tnfα* and *Il1β* gene expression in wounded and unwounded skin tissue. In addition, we found decreased *Col1a1* expression in wounded skin from HFD DM+ mice and increased *Ins1* expression in unwounded skin from 8-month old HFD mice (S1C Fig). In wounded skin from HFD DM+ mice, we also observed increased expression of genes involved in insulin action.

DFU is the most common skin condition associated with diabetes and, because of the lack of optimal therapeutic interventions, it leads to prolonged periods of inactivity, high risk of infections and eventually the need for amputations [38–40]. The development of new therapeutic interventions that could promote faster and more effective wound healing could improve life quality of affected patients, reducing the risk for severe outcomes. However, testing new agents that could improve the therapeutics of DFU is frequently a problem as ethical issues impose a number of restrictions. Thus, at least in early phases of testing, animal models can provide a fast and effective approach to identify candidates with high chance of success. Additionally, after 12 days post wounding, transcriptomic, micro and macro wound evaluations suggest HFD DM+ mice are still resolving inflammatory and proliferative phase. Micro and Macroscopic evaluation showed increased wound area and delayed wound healing rate, altogether, suggesting a HFD pro-inflammatory phenotype and a healing-delayed HFD DM+ phenotype.

Most animal models used to date do not match clinical conditions frequently seen in humans with DM and DFU. Usually mice models with severe diabetes, develop such condition early in life, thus, the important component of aging is missing [12–14]. Conversely, in models of DM induced by the consumption of energy dense diets lead to a chronic condition that can be sustained until late life; however, under these circumstances, metabolic abnormalities are generally mild [41–43].

## Conclusion

Here we present an old age, obesity stressed mouse model with old age onset of diabetes. We showed that mice submitted to a 8 month long physiologic stress developed severe metabolic abnormalities associated with aging, long-term obesity and subsequent onset of diabetes. For example, tissue abnormalities such as hepatic steatosis occurred but the most striking was a skin phenotype which histologically and transcriptionally could be compared to aging DM patients. Thus, this unique model is perhaps useful to therapeutic development for the treatment of human decubitus ulcers.

## Limitation

Since we induced diabetes in 5-month-old HFD mice, we did not have younger diabetic mice for comparison. We used 2- and 5-month-old HFD mice for the HFD DM+ analysis. Further studies will be needed for older HFD DM+ comparison.

## Methods

### RT2 profiler PCR arrays and bulk expression

Gene expression was analyzed as previously described [44]. Briefly, Gene expression was obtained using four different 96-well RT2 Profiler^TM^ PCR Arrays, Mouse Wound Healing (No. PAMM-121Z), Mouse Insulin Pathway (PAMM-030ZC), Mouse Obesity (PAMM-017ZC-12) array and Mouse Aging (PAMM-178ZC) from Qiagen (Maryland, USA), according to the manufacturer’s instructions. For each treatment group and time point, the array was run three times. RT-PCR was run on a StepOnePlus™ Real-Time PCR system (Thermo Fisher Scientific). For data normalization, *Hsp90ab1* and *Gapdh* average was selected as the reference gene based on data from the Mouse Housekeeping Genes RT² Profiler™ PCR Arrays and subsequent calculation of M values using the geNorm software (Biogazelle NV, Gent, Belgium). StepOne Software v.2.3 was used for data analyses, and gene expression was calculated with the ΔΔCt method. A gene was assumed to be differentially expressed if there was at least a twofold difference expression. For skin samples, we used *n* sample = 2 pooling 2–3 animals per sample. For wound healing arrays, we used *n* samples = 3 pooling 2–3 animals per sample. For pancreatic *Ins1&2* mRNA expression, we obtained an *Ins1* and *Ins2* mean expression using *Gapdh* and *Rpl0* mean as endogenous control. For dermal *Lep*, *Ins1*, *Akt1*, *Tnfα*, *Il1β*, and *Il6*, we used *Gapdh* as endogenous control. In Fig 1J we used 2-month old skin samples as reference. In Fig 2I we used skin from Chow diet mice as reference. We used publicly available single cell RNA sequencing datasets as “bulk” expression of diabetic skin from mice and human diabetic. Briefly, Single-cell RNA-seq Pseudobulk gene expressions analysis from mice and human cells were retrieved from diabetic samples [7, 45] (human and mice) according to the gene list of the Mouse Insulin Pathway array (PAMM-030ZC). For Fold change, we used non-diabetic “healthy” skin as reference [7, 46]. As previously described [47], we used *In silico* analyses were performed using a DELL workstation with 16 GB RAM and four-cores Intel i7 processor. Sample expression matrices were downloaded from Gene Expression Omnibus Archive: GSE142471 and GSE165816. Cells were filtered by their total number of reads, by their number of detected genes and by their mitochondrial percentage. For mice, we used nFeature_RNA < 6,000, nCount_RNA < 40,000, percent.mt < 25% settings. For humans, we used nFeature_RNA < 2,500, nCount_RNA < 8,000 and percent.mt < 20% settings. Samples were processed in Seurat v3.1.5 using the default Seurat workflow [48]. For clustering and visualization, we used the default Seurat pipeline and Prism Graphpad (8v) heatmaps.

### Experimental model

Six-week-old male C57BL/6 mice were obtained from the Animal Facility of the University of Campinas. Mice were kept in groups (5 mice per cage) at 21 ± 5°C, in 12/12 h light/dark cycle, with water and chow *ad libitum*. Next, mice were kept in individual cage since 1 week before testing, measurement, and tissue harvest. In all experiments, control and intervention group mice were treated in the same experimental settings. All experiments were conducted according to the “Guide for the Care and Use of Laboratory Animals of the Institute of Laboratory Animal Resources, US National Academy of Sciences” and were approved by the Ethics Committee (CEUA IB/UNICAMP n° 5425-1/2019).

### Dietary interventions

Eight-week-old mice were separated into seven groups. Three groups fed with chow diet (base-line control). The remaining four groups were fed with High-Fat diet (HFD) for 2, 12 or 24 weeks (diets composition in Table 2, experimental design in Fig 1A). Then, mice were subjected to lethal anesthesia and tissues specimens were extracted for analyses.

**Table 2:** Macronutrient component of the diets chow and HFD.

Components	Chow (g)	High-fat diet (g)
Starch	427.5	199.5
Protein (casein 85%)	200	200
Dextrinized corn starch	132	132
Sucrose	100	100
Soyabean oil	40	40
Lard	0	228
Fiber (celulose)	50	50
Mineral mix (ain-93)	35	35
Vitamin mix (ain-93)	10	10
L-cystine	3	3
Choline bitartarate	2.5	2.5
**TOTAL**	**1000**	**1000**

Chow animals were fed with chow diet. HFD and HFD DM+ were fed on HFD diet.

### Diabetes mellitus induction protocol

At 20 weeks of age (5-month old), one group on HFD was induced to diabetes mellitus (DM) with Streptozotocin (STZ). Mice were fasted for 4h before daily intraperitoneal injections of STZ (50 mg/kg). STZ was daily and freshly dissolved in 0.1 M sodium citrate buffer, pH 4.5, and the dose volumes for each mouse were calculated by *Labinsane* app [49] STZ injections were made for five consecutive days (low multiple-dose protocol) [50]. HDF and chow diet were injected sodium citrate buffer as control group.

Induced DM state was assessed after four weeks using an OptiumTM mini (Abbott Diabetes Care, Alameda, CA, USA) handheld glucometer with appropriate test strips. Blood glucose levels were measured by blood from tail vein. Mice exceeded blood glucose levels 300 mg/dL after treatment were considered *diabetic* (HFD DM+).

### Diary food intake and mice weight measurements

To avoid mice distress interference because isolation and tail blood sampling, food and weight measurements were performed one week before ITT and GTT. Before measurements, mice were fasted for 12 hours overnight and then weighed for five consecutive days at the same hour. Food mass and water mass were also weighed at the same time for five consecutive days.

### Tissue extraction

Mice were submitted to fasting according to previously described protocol [51]. Subsequently, mice were anesthetized with lethal doses of xylocaine and ketamine calculated by *Labinsane* App [49]. Pancreas and fragments of dorsal skin (8.0 mm punch), epididymal and white adipose tissue were extracted, weighted and prepared for molecular analysis or histological staining.

#### Glucose and insulin levels analysis

Plasma samples were obtained from fasted mice as here previously described [22]. Whole blood samples collected in EDTA pre-coated tubes, followed by centrifugation (3500 RPM, 15 minutes, room temperature), and were stored at −80°C. Serum glucose was determined by the glucose oxidase method, as previously described [17]. Serum insulin was determined as previously described according to the manufacture’s instruction (Millipore #EZMI-13K) [22].

### Intraperitoneal glucose tolerance test

For Glucose tolerance test (GTT), mice were submitted to fasting protocol according previously described [51]. After 6 h fasting, mice were anesthetized by an intraperitoneal injection of sodium amobarbital (15 mg/kg body weight). Basal glucose concentration was determined from collected tail blood. After collection of an unchallenged sample (time 0), a solution of 20% glucose (2.0 g/kg body weight) was administrated via intraperitoneal and blood from tail vein was collected after 15, 30, 60, 90 and 120 minutes. In both tests, blood glucose concentration was measured using handheld glucometer OptiumTM mini (Abbott Diabetes Care, Alameda, CA, USA) [52].

### Insulin tolerance test

For Insulin tolerance test (ITT) was performed as previously described [22]. Briefly, mice were fasted and tail blood was collected for basal glucose evaluation. Fasted-mice received insulin (1.5 U/kg) by intraperitoneal injection, and blood samples were collected at 0, 5, 10, 15, and 20 min for glucose determination. The rate constant for glucose disappearance during insulin tolerance test (kITT) was calculated using the formula 0.693/t1/2. The glucose t1/2 was calculated from the slope of the least-square analysis of the plasma glucose concentrations during the linear decay phase [52]. Blood glucose concentration was measured using handheld glucometer OptiumTM mini (Abbott Diabetes Care, Alameda, CA, USA).

### Histology

For the histological analyses, skin samples were processed and stained with hematoxylin and eosin as previously described [53]. Briefly, skin and WAT samples were fixed by immersion in paraformaldehyde, processed in alcohol at 70, 80, 95, and 100%, followed by xylol and paraffin, embedded in paraffin blocks. Skin sections (5.0 μm) were placed on microscope slides pretreated with poly-l-lysine. We incubated sections with hematoxylin for 30 s, rinsed in distilled water, incubated for 30 s with eosin, rinsed again in distilled water, and dehydrated [53]. Indirect white adipose tissue in skin sections were determined by the percentage of subcutaneus layer compared to the skin full thickness using H&E skin biopsies in five different animals in triplicate. Indirect hepatic steatosis was determined by the percentage of white ballooning formations in H&E liver biopsies in five different animals in triplicate [54, 55].

### Wound healing documentation and analysis

Full-thickness wounds were made as previously described [56, 57]. Briefly, we used a sterile 6 mm dermal biopsy punch to perform two full-thickness wounds in the back skin of mice. The injury motion consisted of a single, quick perpendicular stroke accompanied by one rotatory movement to free the punch from the skin. The wound cavity was immediately dressed using a transparent bandage (Tegaderm) as previously described [58]. For wound healing analyses, macroscopic evaluation of the healing process was evaluated as previously described [56, 57]. Photographs were taken at 0, 4, 6, 8, 10 and 12 days after wounding using a wide camera 26 mm f/1.6 objective. The same distance (from the wound to the objective lens), top white fluorescence illumination, and operator were used on each occasion. We calculate Wound Area as follows: Wound Area (percentage) = [(wound surface of day 0—wound surface of day x)/wound surface of day 0] × 100. In each group, we use 16 to 20 photographs per day of analysis. Wound healing analysis were examined by ImageJ Software (1.52v) [57].

## Supporting information

S1 Fig Body mass and White adipose tissue mass expressed in grams of chow diet, HFD, and HFD DM+ until 8 months age (a). Daily body mass increase (miligrams per day) of chow diet, HFD, and HFD DM+ until 8 months age (b). mRNA skin expression of Ins1 and Ins2 in chow, HFD and HFD DM+ animals (chow diet animals as reference) (c).
(TIF)
