# Supplementary figures and images for: An older diabetes-induced mice model for studying skin wound healing
Source: PLoS One. 2023 Feb 17;18(2):e0281373. doi: 10.1371/journal.pone.0281373 (PMC9937492; doi:10.1371/journal.pone.0281373)

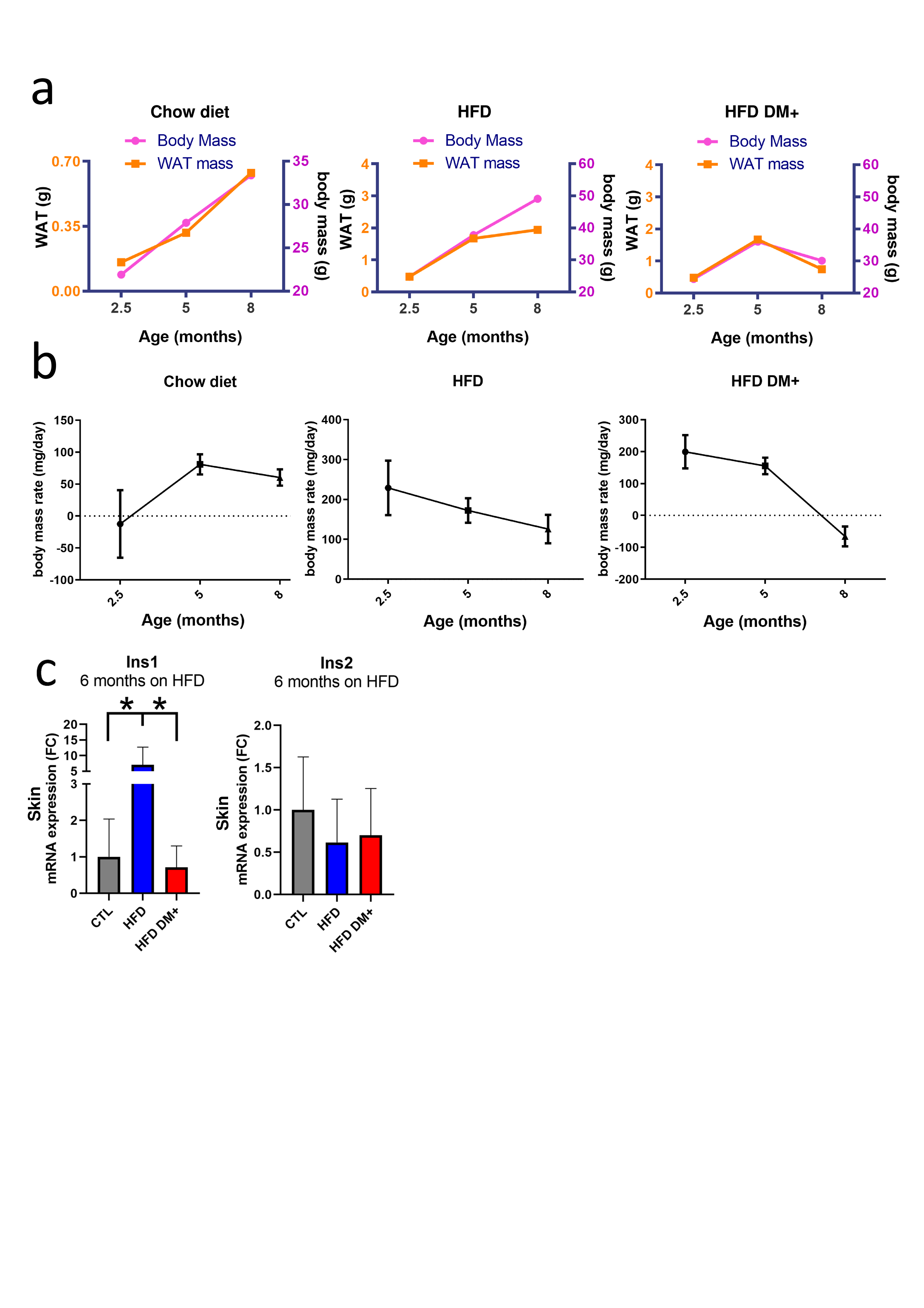

Supplement: S1 Fig — Body mass and White adipose tissue mass expressed in grams of chow diet, HFD, and HFD DM+ until 8 months age (a). Daily body mass increase (miligrams per day) of chow diet, HFD, and HFD DM+ until 8 months age (b). mRNA skin expression of Ins1 and Ins2 in chow, HFD and HFD DM+ animals (chow diet animals as reference) (c). (TIF) [file pone.0281373.s001.tif]
